# Accelerated Brain Aging Identifies Functional Vulnerability Beyond Chronological Age in Multiple Sclerosis

**DOI:** 10.3390/s26082442

**Published:** 2026-04-16

**Authors:** Patrick G. Monaghan, Taylor N. Takla, James H. Cole, Nora E. Fritz

**Affiliations:** 1 Department of Health Care Sciences, Wayne State University, Detroit, MI 48201, USA; 2 Translational Neuroscience Program, Wayne State University, Detroit, MI 48201, USA; 3 Hawkes Institute, Department of Computer Science, University College London, London WC1V 6LJ, UK; 4 Department of Neurology, Wayne State University, Detroit, MI 48201, USA; 5 Institute for Gerontology, Wayne State University, Detroit, MI 48201, USA

**Keywords:** multiple sclerosis, aging, brain age, balance, mobility, physical activity, biomarker

## Abstract

Chronological age incompletely captures neurodegenerative burden and functional vulnerability in multiple sclerosis (MS). Brain-predicted age difference (Brain-PAD; predicted minus chronological age) provides an MRI-derived index of accelerated brain aging, but links to mobility and real-world behavior remain unclear. Forty-three adults with MS completed structural MRI, mobility testing, and six months of free-living physical activity monitoring. Brain age was estimated using PyBrainAge applied to FreeSurfer-derived cortical thickness and subcortical volumes. Hierarchical regressions tested whether Brain-PAD explained additional variance in mobility (Mini-BESTest total and subscores; forward/backward walking velocity) and moderate-to-vigorous physical activity (MVPA) beyond age and disability (PDDS). Predicted brain age exceeded chronological age (Brain-PAD = 8.4 ± 11.1 years; *p* < 0.001). After accounting for age and PDDS, Brain-PAD explained additional variance in Mini-BESTest total (ΔR^2^ = 0.05, *p* = 0.042) and anticipatory control (ΔR^2^ = 0.08, *p* = 0.034), with a trend for sensory orientation. Brain-PAD was not associated with walking velocity beyond PDDS. Higher Brain-PAD was associated with lower MVPA (β = −0.91, *p* = 0.005) and explained additional variance (ΔR^2^ = 0.19). Brain-PAD is elevated in MS and relates to balance control and real-world physical activity beyond age and disability, highlighting its potential to identify functional vulnerability.

## 1. Introduction

Multiple sclerosis (MS) is increasingly recognized as a disease of aging. Global demographic shifts toward older age, coupled with rising prevalence and improved survival in MS, have resulted in a growing proportion of individuals living with MS into later adulthood [1,2,3]. In the United States alone, approximately one million adults live with MS, with more than 40% aged 55 years or older [1]. As a result, many individuals with MS experience the combined burden of aging and chronic neurodegenerative disease, placing them at heightened risk for functional decline and loss of independence.

Mobility impairments are among the most disabling consequences of both aging and MS. Slowed gait and reduced postural stability are common across the disease course and are strongly associated with falls, reduced physical activity, and diminished quality of life [4,5,6]. More than 70% of people with MS report at least one fall over a six-month period, with fall risk closely linked to deficits in gait and balance control [7,8]. Importantly, mobility limitations in MS reflect impairments across multiple, partially dissociable domains of postural control, including anticipatory postural adjustments, reactive balance control, sensory integration, and dynamic gait stability, rather than a single global deficit [9,10]. Different mobility tasks place varying demands on these systems, suggesting that no single assessment fully captures functional vulnerability. Therefore, approaches that integrate complementary measures of walking and balance may provide a more sensitive index of mobility-related risk and better inform early intervention strategies.

Chronological age, while routinely used to contextualize disability risk, provides an incomplete proxy for the biological processes that drive functional decline. In the general population, deterioration of physiological systems accelerates during the sixth and seventh decades of life, contributing to mobility decline [11]. In MS, however, inflammatory and neurodegenerative pathology often begins decades earlier, such that physical function may already be compromised by midlife [12,13]. Emerging evidence supports the concept of accelerated biological aging in MS across multiple systems, including immune, epigenetic, and neuroanatomical markers [14,15,16]. Collectively, this evidence underscores the importance of biomarkers that index aging processes within the central nervous system, where neurodegeneration is most closely linked to mobility and functional decline in MS.

Neuroimaging-derived estimates of brain aging have emerged as a promising biomarker of brain health. Machine-learning models trained on structural MRI data can accurately predict chronological age in healthy individuals; the deviation between predicted brain age and chronological age, referred to as brain-predicted age difference (Brain-PAD), reflects aberrant accumulation of age-related brain changes [17,18,19]. Across neurological and psychiatric conditions, elevated Brain-PAD has been associated with structural brain alterations, disease burden, and adverse clinical outcomes [20,21]. In MS, multiple studies have demonstrated accelerated brain aging relative to chronological age, supporting Brain-PAD as a biologically plausible marker of neurodegenerative burden [17,22,23,24,25,26].

Recent work has further linked Brain-PAD to clinical disability and motor performance in MS, supporting its relevance at the level of impairment [17,25]. However, impairment-level measures provide a global summary of motor function and may fail to capture domain-specific variability in the motor control processes that support balance and gait. Whether MRI-derived brain aging relates to distinct balance subsystems, such as anticipatory control, reactive balance, dynamic balance, and sensory integration assessed during clinical mobility testing, remains largely unexplored. Moreover, the extent to which brain aging predicts how individuals function in their daily environments, beyond laboratory-based assessments, is poorly understood. Real-world physical activity represents a critical functional outcome in MS that provides insight into daily functioning. Free-living activity levels, particularly moderate-to-vigorous physical activity (MVPA), are strongly associated with cardiovascular health, fall risk, and quality of life in this population [27,28]. While laboratory-based mobility assessments provide valuable insight into motor capacity, they may not fully capture the behavioral consequences of neurobiological vulnerability in daily life. Establishing links between Brain-PAD and laboratory assessments of both mobility and real-world physical activity would substantially enhance the clinical relevance of brain aging as a biomarker of functional vulnerability.

Traditional clinical metrics in MS, such as relapse frequency, provide limited insight into the underlying neurodegenerative processes that contribute to long-term functional decline. As a result, there is increasing recognition of the need for more personalized and multidimensional approaches to monitoring disease progression that integrate neurobiological, functional, and behavioral markers [29]. Therefore, the objective of this study was to determine whether Brain-PAD explains variability in laboratory-based mobility performance and real-world physical activity in individuals with MS beyond chronological age and disability status. We hypothesized that higher Brain-PAD would be associated with poorer balance and gait performance and lower levels of physical activity. We further hypothesized that Brain-PAD would explain additional variance in these functional outcomes beyond that accounted for by chronological age and disability status. By linking brain aging to both laboratory-based mobility and real-world physical activity, this work aims to clarify the functional significance of neurodegenerative burden in MS.

## 2. Materials and Methods

### 2.1. Study Population

Participants were eligible for inclusion if they: (1) had a diagnosis of relapsing–remitting MS according to the McDonald criteria; (2) were between 18 and 65 years of age; (3) reported a Patient-Determined Disease Steps (PDDS) score ≤ 6, indicating the ability to ambulate with or without an assistive device for at least 50% of the time; (4) had normal or corrected-to-normal vision; and (5) were able to understand and follow study instructions. Participants were excluded if they: (1) experienced an MS relapse within the eight weeks preceding study participation; (2) had an acute orthopedic condition affecting walking (e.g., recent fracture or sprain); (3) had a comorbid neurologic disorder; (4) used corticosteroids within the previous 30 days; (5) were currently taking dalfampridine (4-aminopyridine), a medication known to improve walking performance; (6) had metallic implants or other contraindications to MRI; or (7) self-reported pregnancy. This set of criteria resulted in a relatively homogenous sample of ambulatory individuals with relapsing-remitting MS and mild-to-moderate disability. While this approach reduces confounding variability, it may limit generalizability to individuals with progressive disease or more advanced disability. The final analytic sample consisted of 43 individuals with MS who completed neuroimaging, laboratory-based mobility assessments, and prospective physical activity monitoring. All study procedures were approved by the Wayne State University Institutional Review Board, and all participants provided written informed consent in accordance with the Declaration of Helsinki.

### 2.2. Experimental Procedures

Participants completed a comprehensive assessment protocol consisting of neuroimaging, laboratory-based clinical mobility testing, and prospective monitoring of free-living physical activity. Structural MRI and clinical assessments were completed during a laboratory visit conducted by trained research staff using standardized protocols. Following the laboratory visit, participants were monitored continuously for physical activity in their daily environments for a six-month period.

### 2.3. Clinical and Behavioral Outcomes

#### 2.3.1. Disability Status

Global disability was assessed using the Patient-Determined Disease Steps (PDDS), a validated self-report measure of MS-related disability [30]. PDDS scores range from 0 to 8, with higher scores indicating greater disability. In the present study, PDDS was treated as a continuous variable and included as a covariate in all regression analyses to account for overall disease-related functional impairment.

#### 2.3.2. Laboratory-Based Mobility Outcomes

Balance and postural control were assessed using the Mini-Balance Evaluation Systems Test (Mini-BESTest), a validated clinical assessment designed to evaluate distinct components of balance control [31]. The Mini-BESTest yields a total score as well as subscores reflecting anticipatory postural control, reactive postural control, sensory orientation, and dynamic gait stability. These outcomes were examined separately to assess whether brain aging was differentially associated with specific balance subsystems.

Gait performance was assessed using the Timed 25-Foot Walk Test performed in both the forward and backward directions. Participants completed two trials in each direction at a self-selected comfortable walking speed. For each trial, participants began with both feet positioned behind the starting line and were instructed to walk continuously to the end of the walkway. Timing commenced when the first foot crossed the starting line and ceased when the first foot crossed the finish line. Completion times from the two trials were averaged separately for forward and backward walking and subsequently converted to walking velocity (m/s). Both forward and backward versions of the Timed 25-Foot Walk Test have demonstrated strong reliability and validity in individuals with multiple sclerosis and are commonly used to quantify locomotor performance under varying task demands [32,33].

#### 2.3.3. Real-World Physical Activity Outcomes

Free-living physical activity was assessed prospectively using a wrist-worn activity monitor (Fitbit Versa) over a six-month monitoring period. The device incorporates a triaxial accelerometer and optical heart rate sensor to estimate daily step counts and classify activity intensity, including moderate-to-vigorous physical activity (MVPA) [34,35]. Physical activity data were collected and managed using the Fitabase platform (Fitabase, San Diego, CA, USA). Participants were instructed to wear the device during all waking hours and to maintain their usual daily activities throughout the monitoring period. Primary physical activity outcomes included average daily MVPA, with secondary exploratory outcomes including total daily step counts and sedentary minutes. Consistent with prior work, a minimum of 27 valid wear days was required to ensure stable estimation of MVPA and to exceed established thresholds for step counts and sedentary behavior [36]. A valid day was defined as one with at least 10 h of wear time [37]. Wrist-worn activity monitors have demonstrated good feasibility and validity for measuring physical activity in individuals with multiple sclerosis [38]. The wrist-based configuration was selected to minimize participant burden and maximize long-term adherence during extended monitoring periods [39].

### 2.4. MRI Acquisition and Processing

Structural MRI data were acquired during the laboratory visit using a 3T Siemens MAGNETOM Verio scanner (Siemens Healthineers, Erlangen, Germany) equipped with a 32-channel head coil. High-resolution T1-weighted images were collected using a three-dimensional multi-echo magnetization-prepared rapid gradient echo (MEMPRAGE) sequence (176 sagittal slices; 1.0 mm isotropic resolution; field of view = 256 mm; repetition time = 2530 ms; flip angle = 7°; GRAPPA acceleration factor = 2; echo times = 1.79, 3.65, 5.51, and 7.37 ms).

Structural images were visually inspected for motion artifacts and gross image distortions prior to processing. T1-weighted images passing quality control were processed using FreeSurfer (version 7.3.2; Martinos Center for Biomedical Imaging, Harvard Medical School) to extract cortical thickness and subcortical volumetric measures required for brain age estimation. Cortical thickness measures were derived from the Destrieux atlas, and subcortical volumes were extracted using standard FreeSurfer segmentation procedures. These measures were used solely as input features for brain age prediction and were not analyzed independently in this study.

### 2.5. Brain Age Prediction

Brain age was estimated using PyBrainAge, an open-source brain age prediction framework implemented in Python v3.7 (https://github.com/james-cole/PyBrainAge; accessed on 21 February 2025). PyBrainAge estimates chronological age from FreeSurfer-derived features derived from structural MRI scans using machine-learning regression.

Neuroimaging input features consisted of cortical thickness and subcortical volumetric measures extracted from FreeSurfer outputs based on the Destrieux atlas parcellations. Specifically, features were generated using FreeSurfer’s aparcstats2table and asegstats2table functions, resulting in a total of 187 neuroanatomical features per participant. These features were organized into a standardized input format consistent with the PyBrainAge model requirements.

Brain age was predicted using a pre-trained Extra Trees regression model that was originally trained on a large sample of healthy individuals spanning a wide age range. The PyBrainAge model was trained on a large multi-site dataset of healthy individuals, as described in Rutherford et al. [40]. While technical variability across MRI scanners and acquisition protocols can influence brain age estimates, prior work suggests high within-scanner repeatability but more variable between-scanner reproducibility, and these factors should be considered when interpreting brain-PAD values [41]. Brain-PAD was calculated as predicted brain age minus chronological age, such that positive values reflected older-appearing brains relative to chronological age. Brain-PAD was treated as a continuous index of neurodegenerative burden in all analyses.

All brain age estimates were inspected for plausibility, and internal consistency was verified by confirming that Brain-PAD values reflected the arithmetic difference between predicted brain age and chronological age for each participant.

### 2.6. Statistical Analysis

All statistical analyses were conducted using R (version 4.5.1). Descriptive statistics were computed for demographic, clinical, neuroimaging, mobility, and physical activity variables. Prior to analysis, all numeric variables were inspected for data integrity, missingness, and plausible ranges. Continuous variables were examined for normality using histograms, skewness, kurtosis, and boxplots. Brain-PAD was further inspected for extreme values using standardized z-scores, with no observations exceeding ±3 standard deviations.

To test whether predicted brain age exceeded chronological age in individuals with MS, a paired-samples *t*-test was performed comparing predicted brain age and chronological age. A directional hypothesis was specified, testing whether predicted brain age was greater than chronological age. Effect size was quantified using Cohen’s d for paired samples, with a small-sample correction applied to estimate Hedges’ g.

Pearson correlation analyses were used to examine associations between Brain-PAD, chronological age, disability status (PDDS), symptom duration, and laboratory-based mobility outcomes. Correlations were computed using pairwise deletion to maximize available data. These analyses were considered exploratory and used to inform subsequent regression models.

Hierarchical linear regression models were used to examine whether Brain-PAD explained variability in laboratory-based mobility outcomes beyond chronological age and disability status. Separate models were constructed for Mini-BESTest total score, Mini-BESTest subscores (anticipatory postural control, sensory orientation, and dynamic gait), forward comfortable walking velocity, and backward comfortable walking velocity. For each outcome, predictors were entered in a stepwise manner: chronological age was entered in the first step, PDDS was added in the second step, and Brain-PAD was entered in the final step. Model improvement associated with the inclusion of Brain-PAD was evaluated using changes in explained variance (ΔR^2^) and corresponding F-tests from nested model comparisons. Regression coefficients from the full models were examined to determine the direction and magnitude of associations.

Hierarchical linear regression was similarly used to examine associations between Brain-PAD and real-world physical activity outcomes derived from six months of accelerometer monitoring. Separate models were constructed for average daily moderate-to-vigorous physical activity (MVPA), total daily steps, and sedentary minutes. As with laboratory-based outcomes, chronological age was entered in the first step, PDDS in the second step, and Brain-PAD in the final step. Incremental variance explained by Brain-PAD was quantified using ΔR^2^, and regression coefficients from the final models were interpreted to assess associations with real-world behavior. As a sensitivity analysis, hierarchical regression models additionally included normalized brain volume to determine whether associations between Brain-PAD and outcomes were independent of global brain atrophy (see Supplementary Materials).

## 3. Results

### 3.1. Participant Characteristics

The final analytic sample included 43 individuals with relapsing–remitting multiple sclerosis who completed structural neuroimaging, laboratory-based mobility testing, and prospective physical activity monitoring. Participants were 47.8 ± 10.0 years old (range: 31–65) with a mean symptom duration of 15.3 ± 8.5 years (range: 2–39). Disability severity was generally mild-to-moderate (PDDS range: 0–6; M = 1.9 ± 2.0). Predicted brain age demonstrated substantial interindividual variability (M = 56.2 ± 12.3 years), corresponding to a mean Brain-PAD of 8.43 ± 11.08 years (median = 7.91; range = −13.56 to 41.56). Brain-PAD values were approximately normally distributed (skewness = 0.30, kurtosis = 0.26), with no observations exceeding ±3 SD (z-score range: −1.98 to 2.99). Descriptive statistics for all study variables are presented in Table 1.

### 3.2. Brain Age and Brain-PAD in MS

**Figure 1:**
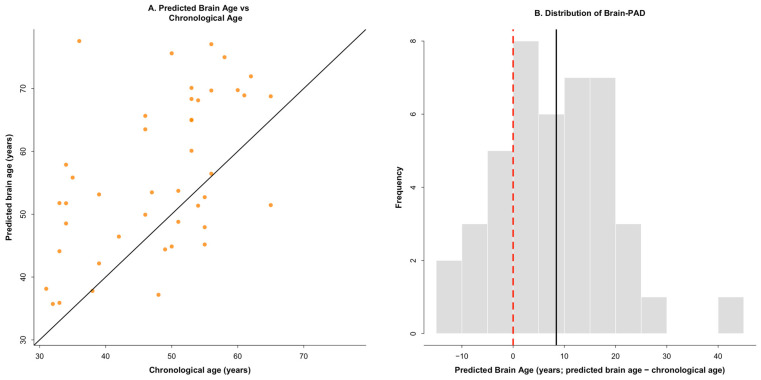
Brain age and brain-predicted age difference (Brain-PAD) in individuals with multiple sclerosis. (**A**) Scatterplot showing the relationship between chronological age and predicted brain age. Each point represents an individual participant. The solid diagonal line indicates the line of identity (predicted brain age = chronological age), such that points above the line reflect older-appearing brains relative to chronological age. (**B**) Histogram illustrating the distribution of brain-predicted age difference (Brain-PAD; predicted brain age minus chronological age). The dashed red vertical line denotes Brain-PAD = 0 (no apparent brain aging acceleration), whereas the solid black vertical line indicates the sample mean Brain-PAD (mean = 8.4 years). Positive Brain-PAD values reflect accelerated brain aging relative to chronological age.

Predicted brain age was significantly greater than chronological age in the MS cohort. On average, predicted brain age exceeded chronological age by 8.43 ± 11.08 years. A paired-samples *t*-test indicated that predicted brain age was higher than chronological age, t(42) = 4.99, *p* < 0.001, with a moderate-to-large effect (Cohen’s d = 0.76; Hedges’ g = 0.75). Brain-PAD values were predominantly positive and demonstrated substantial interindividual variability (range = −13.56 to 41.56), indicating heterogeneity in the degree of accelerated brain aging (Figure 1).

### 3.3. Associations Between Brain-PAD, Disability, and Laboratory-Based Mobility

Pearson correlation analyses examined bivariate associations among Brain-PAD, chronological age, disability status (PDDS), and laboratory-based mobility outcomes (Table 2). Higher Brain-PAD was modestly associated with poorer overall balance performance, as reflected by lower Mini-BESTest total scores (r = −0.29, *p* = 0.058), with a statistically significant association observed for the anticipatory postural control subscore (r = −0.31, *p* = 0.044). Similar associations were observed for the sensory orientation subscore (r = −0.29, *p* = 0.055), although this did not reach conventional significance. In contrast, Brain-PAD was not associated with reactive postural control (r = −0.11, *p* = 0.476). Associations between Brain-PAD and dynamic gait subscores were weaker and non-significant (r = −0.24, *p* = 0.125).

Brain-PAD was not significantly correlated with forward comfortable walking velocity (r = −0.22, *p* = 0.147) or backward comfortable walking velocity (r = −0.15, *p* = 0.335). Chronological age showed no significant associations with Mini-BESTest total score, subscores, or gait velocity outcomes (all r ≤ 0.15, all *p* ≥ 0.33).

Higher disability severity was strongly associated with poorer mobility. PDDS was negatively correlated with Mini-BESTest total score (r = −0.72, *p* < 0.001), anticipatory (r = −0.54, *p* < 0.001), sensory (r = −0.54, *p* < 0.001), and dynamic gait subscores (r = −0.74, *p* < 0.001), as well as forward (r = −0.62, *p* < 0.001) and backward walking velocity (r = −0.61, *p* < 0.001). These findings motivated subsequent regression analyses to determine whether Brain-PAD explained unique variance in mobility outcomes beyond age and disability.

### 3.4. Brain-PAD and Laboratory-Based Mobility Outcomes

Hierarchical linear regression models tested whether Brain-PAD explained variability in laboratory-based mobility outcomes beyond chronological age and PDDS. Chronological age was entered at Step 1, PDDS at Step 2, and Brain-PAD at Step 3. Incremental variance explained by Brain-PAD was evaluated using ΔR^2^ and nested F-tests, and coefficients from the final models were examined (Table 3).

#### 3.4.1. Balance Performance (Mini-BESTest)

For Mini-BESTest total score, age explained minimal variance (R^2^ = 0.02), whereas adding PDDS substantially increased explained variance (ΔR^2^ = 0.50, *p* < 0.001). Brain-PAD explained additional variance beyond age and PDDS (ΔR^2^ = 0.05, F(1, 39) = 4.44, *p* = 0.042). In the final model, higher Brain-PAD was independently associated with lower total scores (β = −0.11, *p* = 0.042), whereas age was not significant (*p* = 0.23).

A similar pattern was observed for anticipatory control. Brain-PAD explained additional variance beyond age and PDDS (ΔR^2^ = 0.08, F(1, 39) = 4.82, *p* = 0.034), with higher Brain-PAD associated with poorer anticipatory performance (β = −0.04, *p* = 0.034). For sensory orientation, Brain-PAD contributed variance at trend level (ΔR^2^ = 0.06, *p* = 0.055). In contrast, Brain-PAD did not explain additional variance in reactive postural control (posture subscore; ΔR^2^ = 0.01, *p* = 0.572) or dynamic gait performance (ΔR^2^ = 0.02, *p* = 0.18). Across balance models, PDDS was consistently associated with poorer performance (*p* ≤ 0.016), whereas chronological age was not significant.

#### 3.4.2. Gait Velocity

For both forward and backward comfortable walking velocity, age explained negligible variance. PDDS significantly increased explained variance for forward (ΔR^2^ = 0.39, *p* < 0.001) and backward walking velocity (ΔR^2^ = 0.37, *p* < 0.001). Brain-PAD did not explain additional variance in forward (ΔR^2^ = 0.01, *p* = 0.40) or backward walking velocity (ΔR^2^ < 0.01, *p* = 0.62). In the final models, higher PDDS was associated with slower walking speed in both directions (both *p* < 0.001).

### 3.5. Brain-PAD and Physical Activity

Hierarchical regression models examined whether Brain-PAD explained variability in real-world physical activity outcomes beyond age and PDDS (Table 3). For MVPA, age (R^2^ = 0.003) and PDDS (ΔR^2^ = 0.005, *p* = 0.65) explained minimal variance, whereas adding Brain-PAD significantly increased explained variance (ΔR^2^ = 0.19, F(1, 37) = 8.86, *p* = 0.005). In the final model, higher Brain-PAD was independently associated with lower MVPA (β = −0.91, SE = 0.31, *p* = 0.005), whereas age and PDDS were not significant (both *p* > 0.59). In sensitivity analyses additionally adjusting for normalized brain volume, Brain-PAD remained independently associated with MVPA (ΔR^2^ = 0.23, *p* = 0.002; Supplementary Table S2), indicating that this association was not attributable to global brain atrophy alone.

For total daily steps, PDDS significantly increased explained variance at Step 2 (ΔR^2^ = 0.11, *p* = 0.038), whereas Brain-PAD did not explain additional variance (ΔR^2^ = 0.02, *p* = 0.37). In the full model, the PDDS coefficient was attenuated to trend level (β = −420 steps/day, *p* = 0.061). For sedentary time, PDDS accounted for substantial variance (ΔR^2^ = 0.29, *p* < 0.001), whereas Brain-PAD did not improve model fit (ΔR^2^ = 0.02, *p* = 0.32). Greater disability was associated with increased sedentary time (β = 29.8 min/day, *p* < 0.001).

## 4. Discussion

In this study, we examined whether MRI-derived brain aging provides insight into functional vulnerability in MS beyond chronological age and global disability status. Brain-PAD was significantly elevated in our cohort, with predicted brain age exceeding chronological age by approximately 8 years on average, consistent with accelerated neurobiological aging in MS. Recent work has similarly demonstrated elevated Brain-PAD in large MS cohorts, supporting the utility of brain age as a sensitive marker of neurodegenerative burden [25,42,43]. Importantly, Brain-PAD explained unique variance in balance performance, particularly Mini-BESTest total score and the anticipatory domain, after accounting for chronological age and PDDS, whereas chronological age showed minimal associations with laboratory-based mobility. Extending these findings to real-world behavior, higher Brain-PAD was robustly associated with lower daily moderate-to-vigorous physical activity (MVPA) over six months, explaining 19% of MVPA variance, and again outperforming both chronological age and disability status, neither of which meaningfully predicted physical activity levels. Together, these results suggest that accelerated brain aging captures meaningful heterogeneity in functional vulnerability in MS, linking neurodegenerative burden to both multi-domain balance control and everyday physical activity behavior.

The selective association between Brain-PAD and specific balance subsystems suggests that accelerated brain aging preferentially affects motor control processes relying on prediction and multisensory integration. Anticipatory postural control depends on feedforward mechanisms integrating cortical planning, subcortical coordination, and sensorimotor prediction, while sensory orientation reflects the ability to reweight visual, vestibular, and somatosensory inputs under changing task demands [44,45]. Both processes show age-related decline in healthy adults, with deterioration beginning in midlife and progressing across the lifespan [46,47]. Sensory reweighting becomes less adaptive with aging, with older adults demonstrating increased visual dependence and reduced capacity to rapidly reweight vestibular information during sensory conflict [48,49]. The present findings extend this literature to MS by demonstrating that MRI-derived brain aging captures heterogeneity in these higher-order balance systems beyond chronological age or global disability. Notably, impaired anticipatory postural control and sensory orientation are associated with functional task limitations in Parkinson’s disease, including difficulties with sit-to-stand and step-up-and-over tasks, suggesting these domains may be sensitive indicators of real-world mobility constraints [50]. In contrast, reactive postural responses and gait velocity may be more strongly constrained by peripheral strength or reflexive pathways, rendering them less sensitive to subtle neurobiological aging once disability is accounted for. This pattern supports the interpretation that Brain-PAD reflects aging-related degradation of complex, multisensory balance systems rather than generalized motor impairment.

Beyond laboratory-based mobility, Brain-PAD demonstrated a robust association with real-world physical activity, with higher brain aging predicting lower daily moderate-to-vigorous physical activity over six months. This relationship is particularly striking given that it persisted after accounting for chronological age and disability status, neither of which meaningfully explained variance in MVPA. Real-world physical activity reflects a complex behavioral outcome integrating motor capacity with motivation, confidence, fatigue, cognitive resources, and environmental demands, and may be particularly sensitive to diffuse neurobiological vulnerability captured by MRI-derived brain aging. Objective accelerometry studies demonstrate that MVPA declines progressively across the adult lifespan, with device-measured sedentary behavior and physical activity patterns differing substantially by demographic and health-related factors in older adults [51,52]. Recent latent profile analyses have identified distinct physical behavior phenotypes, with inactive profiles characterized by lower activity volume, higher fragmentation, and reduced physical capacity, patterns that may reflect underlying neurobiological vulnerability [53]. Importantly, maintaining higher MVPA levels may offset cardiovascular risk in older adults, with evidence demonstrating that activity intensity and sedentary break patterns, rather than sedentary time alone, are critical determinants of cardiometabolic health [54,55]. The present study extends prior work linking Brain-PAD to impairment-level outcomes by demonstrating that accelerated brain aging is associated with meaningful reductions in everyday behavior, providing evidence that brain aging relates not only to how individuals perform in structured testing environments but also to how they function in daily life [17,42].

These findings suggest that MRI-derived brain aging may have clinical utility for identifying individuals with MS who are functionally vulnerable despite relatively preserved chronological age or disability status. Brain-PAD may help detect neurobiological risk before overt declines in mobility or physical activity become clinically evident, supporting earlier or more targeted intervention strategies such as proactive referral to physical therapy, implementation of balance training programs, or consideration of neuroprotective therapies. An important observation from the present data is the substantial heterogeneity in real-world physical activity at a given level of brain aging, suggesting that certain factors may mitigate the functional consequences of accelerated neurobiological aging. Recent work in aging populations demonstrates that physical activity is associated with younger-appearing brains and may protect against biological brain aging [56], suggesting a potential bidirectional relationship whereby habitual physical activity influences Brain-PAD trajectories. While the present study was not designed to test these moderators directly, prior work suggests that cognitive reserve gained through intellectual enrichment [57,58], lower cardiovascular risk burden, and higher habitual physical activity [59] may confer resilience against functional decline in both aging populations and MS. Importantly, both brain reserve (maximal lifetime brain volume) and cognitive reserve (intellectual enrichment) have been shown to independently protect against disease-related cognitive and functional decline in MS [60,61], with lifestyle factors such as intellectual engagement demonstrating protective effects even after accounting for genetic contributions. Whether similar mechanisms might buffer the impact of Brain-PAD on mobility and daily function represents an important avenue for future investigation. Framed this way, Brain-PAD may serve not only as a marker of risk but also as a tool to guide personalized prevention and rehabilitation strategies, identifying individuals who may benefit most from targeted interventions to preserve mobility and independence. Notably, these associations emerged in a relatively young, mildly affected sample (mean age 48 years; mean PDDS 1.9), suggesting that Brain-PAD captures meaningful heterogeneity even before substantial disability accumulation. This raises the intriguing question of whether associations between Brain-PAD and functional outcomes would be amplified in older or more clinically diverse cohorts. Future work in samples spanning a wider age range and disease severity spectrum will be critical to understanding how the functional relevance of brain aging may vary across the MS disease course.

Several limitations should be considered when interpreting these findings. First, the cross-sectional design precludes causal inference; longitudinal studies tracking concurrent changes in Brain-PAD and functional outcomes are needed to establish temporal precedence. Second, the study did not include a healthy control group. However, the use of a normative brain age model trained on large healthy cohorts and the demonstration that Brain-PAD explains unique variance beyond chronological age provides meaningful context. Recent work has established that Brain-PAD is elevated in MS relative to healthy controls and predicts disability progression, supporting the clinical relevance of our findings [17,25,42]. Third, the modest sample (N=43) consisted of middle-aged individuals with relapsing-remitting MS and mild-to-moderate disability, resulting in a relatively narrow clinical profile. Given the substantial heterogeneity of MS across disease phenotypes, disability levels, and comorbid conditions, these findings may not generalize to individuals with progressive MS, greater disability, or more complex clinical presentation. Accordingly, the broader clinical applicability of Brain-PAD will require validation in larger and more diverse cohorts spanning the full spectrum of disease severity. Importantly, however, detecting meaningful associations in this relatively young, mildly affected sample suggests Brain-PAD captures vulnerability even before substantial disability accumulates, precisely when early risk identification has greatest preventive value. Additionally, we did not include MRI-derived measures of disease activity (e.g., T2 lesion burden or Gadolinium-enhancing lesions), which may influence neurodegeneration and functional outcomes. Future studies integrating brain age metrics with conventional markers of inflammatory disease activity will be important to better contextualize these relationships. We also did not include disease duration or treatment-related factors, such as disease-modifying therapy use, as covariates in the analytic models, which may influence neurodegenerative processes and functional outcomes in MS. Finally, unmeasured contextual factors (e.g., environmental constraints, psychosocial variables, comorbidities, and fatigue) may have influenced the six-month physical activity monitoring, particularly given that wrist-worn devices rely on proprietary algorithms that may introduce measurement error (e.g., under- or overestimation due to arm movement variation), although they provide feasible and ecologically valid estimates of real-world behavior [62]. Despite these limitations, this study is among the first to link MRI-derived brain aging to both subsystem-level balance control and objectively measured real-world physical activity in MS.

## 5. Conclusions

In summary, this study demonstrates that MRI-derived brain aging is elevated in MS and is more closely linked to subsystem-level balance control and real-world physical activity than chronological age or disability status alone. The selective associations with anticipatory postural control and sensory orientation, balance domains that decline with aging in healthy populations and predict functional limitations in daily life, suggest that Brain-PAD may capture early markers of aging-related motor vulnerability. By connecting neurobiological aging to both laboratory-based motor function and everyday behavior, these findings position Brain-PAD as a sensitive marker of functional vulnerability that may enable earlier identification of individuals at risk for mobility decline and inform personalized timing of rehabilitation and neuroprotective strategies.

## Figures and Tables

**Table 1:** Participant Characteristics.

Variable	Mean (SD)	Median (Range)
**Demographics and Clinical Characteristics**		
Chronological Age, years	47.77 (9.98)	50.0 (31–65)
Sex, female, n (%)	34 (79.1%)	—
Symptom duration, years	15.26 (8.53)	14.0 (2–39)
PDDS score	1.91 (2.03)	1.0 (0–6)
Disease-modifying therapy use, n (%)	24 (55.8%)	—
**Age Metrics**		
Predicted brain age, years	56.20 (12.33)	53.47 (35.72–77.56)
Brain-PAD, years	8.43 (11.08)	7.91 (−13.56–41.56)
**Laboratory-Based Mobility**		
Mini-BESTest total score (max 28)	21.21 (5.26)	23 (2–27)
Anticipatory subscore (max 6)	4.44 (1.47)	5 (0–6)
Reactive Postural Control (max 6)	3.81 (1.30)	4 (0-6)
Sensory subscore (max 6)	5.49 (1.18)	6 (0–6)
Dynamic gait subscore (max 10)	7.47 (2.47)	8 (1–10)
Forward walking velocity, m/s	0.98 (0.32)	1.02 (0.14–1.75)
Backward walking velocity, m/s	0.69 (0.30)	0.74 (0.06–1.28)
**Physical Activity (6 months)**		
MVPA, min/day	21.97 (20.01)	14.51 (0.66–80.58)
Total steps, steps/day	6075 (3026)	6000 (968–12,695)
Sedentary time, min/day	652.0 (101.9)	629.4 (496–1019)

**Note.** Values are presented as mean (standard deviation) unless otherwise indicated. PDDS = Patient-Determined Disease Steps; Brain-PAD = brain-predicted age difference (predicted brain age − chronological age); Mini-BESTest = Mini-Balance Evaluation Systems Test; MVPA = moderate-to-vigorous physical activity. MVPA, total steps, and sedentary time reflect average daily values derived from six months of accelerometer monitoring. Two participants were excluded from physical activity analyses due to insufficient wear time.

**Table 2:** Pearson correlations among Brain-PAD, chronological age, disability, and laboratory-based mobility outcomes (N = 43).

Variable	1	2	3	4	5	6	7	8	9	10
1. Brain-PAD	—									
2. Age	−0.32 *	—								
3. PDDS	0.15	0.13	—							
4. Mini-BESTest Total	−0.29	−0.15	−0.72 ***	—						
5. Mini-BESTest Anticipatory	−0.31 *	−0.15	−0.54 ***	0.80 ***	—					
6. Mini-BESTest Reactive	−0.11	−0.12	−0.40 **	0.66 ***	0.33 *	—				
7. Mini-BESTest Sensory	−0.29	−0.13	−0.54 ***	0.87 ***	0.70 ***	0.60 ***	—			
8. Mini-BESTest Dynamic Gait	−0.24	−0.10	−0.74 ***	0.89 ***	0.61 ***	0.40 **	0.65 ***	—		
9. Forward Walking Velocity	−0.22	0.03	−0.62 ***	0.76 ***	0.54 ***	0.61 ***	0.55 ***	0.72 ***	—	
10. Backward Walking Velocity	−0.15	−0.07	−0.61 ***	0.76 ***	0.58 ***	0.58 ***	0.55 ***	0.71 ***	0.90 ***	—

**Note.** Values are Pearson correlation coefficients (*r*). Brain-PAD = brain-predicted age minus chronological age; PDDS = Patient-Determined Disease Steps. Walking velocities are reported in m/s. *p* values are two-tailed. *p* < 0.05 *, *p* < 0.01 **, *p* < 0.001 ***.

**Table 3:** Hierarchical Linear Regression Models Examining Associations Between Brain-PAD and Mobility and Physical Activity Outcomes.

**A. Laboratory-Based Mobility Outcomes**								
**Outcome**	**Step**	**Predictors**	**β**	**SE**	**t**	***p***	**R^2^**	**ΔR^2^**
**Mini-BESTest Total**	1	Age	−0.07	0.06	−1.23	0.226	0.02	—
	2	+ PDDS	−1.72	0.28	−6.13	<0.001 ***	0.52	0.50 ***
	3	+ Brain-PAD	−0.11	0.05	−2.11	0.042 *	0.57	0.05 *
**Mini-BESTest Anticipatory**	1	Age	−0.03	0.02	−1.37	0.178	0.02	—
	2	+ PDDS	−0.34	0.09	−3.60	<0.001 ***	0.29	0.27 ***
	3	+ Brain-PAD	−0.04	0.02	−2.20	0.034 *	0.37	0.08 *
**Mini-BESTest Reactive**	1	Age	−0.01	0.02	−0.66	0.517	0.02	—
	2	+ PDDS	−0.24	0.10	−2.53	0.016 *	0.17	0.15 *
	3	+ Brain-PAD	−0.01	0.02	−0.57	0.572	0.18	0.01
**Mini-BESTest Sensory**	1	Age	−0.02	0.02	−1.11	0.276	0.02	—
	2	+ PDDS	−0.28	0.08	−3.60	<0.001 ***	0.29	0.27 ***
	3	+ Brain-PAD	−0.03	0.01	−1.98	0.055	0.35	0.06
**Mini-BESTest Dynamic Gait**	1	Age	−0.01	0.03	−0.52	0.609	0.01	—
	2	+ PDDS	−0.86	0.13	−6.56	<0.001 ***	0.55	0.54 ***
	3	+ Brain-PAD	−0.03	0.03	−1.35	0.185	0.57	0.02
**B. Gait Velocity Outcomes**								
**Outcome**	**Step**	**Predictors**	**β**	**SE**	**t**	***p***	**R^2^**	**ΔR^2^**
**Forward Walking Velocity**	1	Age	0.003	0.005	0.53	0.602	0.001	—
	2	+ PDDS	−0.11	0.02	−4.79	<0.001 ***	0.39	0.39 ***
	3	+ Brain-PAD	−0.004	0.004	−0.86	0.396	0.40	0.01
**Backward Walking Velocity**	1	Age	−0.004	0.004	−0.10	0.920	0.005	—
	2	+ PDDS	−0.10	0.02	−4.65	<0.001 ***	0.38	0.37 ***
	3	+ Brain-PAD	−0.002	0.004	−0.50	0.622	0.38	<0.01
**C. Real-World Physical Activity Outcomes**								
**Outcome**	**Step**	**Predictors**	**β**	**SE**	**t**	***p***	**R^2^**	**ΔR^2^**
**MVPA (min/day)**	1	Age	−0.19	0.35	−0.54	0.593	0.003	—
	2	+ PDDS	0.21	1.57	0.13	0.894	0.008	0.005
	3	+ Brain-PAD	−0.91	0.31	−2.98	0.005 **	0.20	0.19 **
**Total Steps (steps/day)**	1	Age	−52.3	48.1	−1.09	0.284	0.023	—
	2	+ PDDS	−420	217	−1.93	0.061	0.13	0.107
	3	+ Brain-PAD	−38	42	−0.91	0.370	0.149	0.019
**Sedentary Minutes (min/day)**	1	Age	0.74	1.78	0.42	0.679	0.003	—
	2	+ PDDS	29.8	8.02	3.72	<0.001 ***	0.298	0.29 ***
	3	+ Brain-PAD	1.55	1.55	1.01	0.324	0.31	0.02

**Note.** β values are unstandardized regression coefficients. Brain-PAD = brain-predicted age minus chronological age; PDDS = Patient-Determined Disease Steps; MVPA = moderate-to-vigorous physical activity. ΔR^2^ reflects the incremental variance explained by adding predictors at each step. *p* values are two-tailed. *p* < 0.05 *, *p* < 0.01 **, *p* < 0.001 ***.

## Data Availability

The data presented in this study are available upon request from the corresponding author.

## Supplementary Materials

The following supporting information can be downloaded at: https://www.mdpi.com/article/10.3390/s26082442/s1, Supplementary Methods: Calculation of Normalized Brain Volume; Table S1: Spearman correlations among age, brain age metrics, normalized brain volume, and moderate-to-vigorous physical activity (MVPA); Table S2: Hierarchical regression predicting 6-month MVPA including normalized brain volume.

## Abbreviations

The following abbreviations are used in this manuscript:

MS	multiple sclerosis
Brain-PAD	brain-predicted age difference
MRI	magnetic resonance imaging
PDDS	patient-determined disease steps
MVPA	moderate–vigorous physical activity
