# Supplementary material for: Accelerated Brain Aging Identifies Functional Vulnerability Beyond Chronological Age in Multiple Sclerosis
Source: Sensors (Basel). 2026 Apr 16;26(8):2442. doi: 10.3390/s26082442 (PMC13119819; doi:10.3390/s26082442)
Supplement: Supplementary file 1 [file sensors-26-02442-s001.zip › sensors-4191898-supplementary.pdf]

SUPPLEMENTARY MATERIAL

Supplementary Methods

1. Calculation of Normalized Brain Volume
Normalized brain volume (NBV) was calculated to index global brain atrophy adjusted for head size. Supratentorial brain volume (excluding ventricular CSF) was divided by estimated total intracranial volume (eTIV), both derived from FreeSurfer (version 7.3.2; Martinos Center for Biomedical Imaging, Harvard Medical School) automated segmentation of T1-weighted structural MRI scans. This ratio provides a head-size-corrected measure of global brain volume and is commonly used as a proxy of whole-brain atrophy.

2. Supplementary Table S1
Spearman correlations among age, brain age metrics, normalized brain volume, and moderate-to-vigorous physical activity (MVPA).

**Variable**	Age	BrainAge	BrainPAD	Normalized Brain Volume	6-Month MVPA
Age	—				
BrainAge	.538**	—			
BrainPAD	−.300*	.642**	—		
Normalized Brain Volume	−.288*	−.721**	−.504**	—	
6-Month MVPA	.058	−.393*	−.440**	.094	—

Note. N = 41–43 depending on variable availability.

- p < .05, ** p < .01.

3. Supplementary Table S2
Hierarchical Regression Predicting MVPA Including Normalized Brain Volume Hierarchical linear regression models examined whether Brain-PAD explained variance in 6-month MVPA beyond age, disability (PDDS), and normalized brain volume. Chronological age was entered at Step 1, PDDS at Step 2, normalized brain volume at Step 3, and Brain-PAD at Step 4.

Model Fit

**Step**	**Predictors Added**	**R^2**	**ΔR^2**	**Sig. ΔR^2**
1	Age	.003	—	.718
2	+ PDDS	.008	.005	.676
3	+ Normalized Brain Volume	.020	.012	.511
4	+ BrainPAD	.250	.230	.002

Final Model

Predictor	B	SE	β	p	95% CI
Age	−0.519	0.403	−.232	.207	−1.337 to 0.299
PDDS	0.237	1.545	.023	.879	−2.897 to 3.371
Normalized Brain Volume	−85.380	55.152	−.306	.130	−197.233 to 26.473
BrainPAD	−1.294	0.390	−.667	.002	−2.084 to −0.504

Model statistics:

$R^2 = .250$, Adjusted $R^2 = .166$, $F(4,36) = 2.994$, $p = .031$

Collinearity diagnostics:

All variance inflation factors (VIFs) < 2.0.

Brain-PAD remained a significant independent predictor of MVPA after accounting for normalized brain volume.
